# High-resolution processing and sigmoid fusion modules for efficient detection of small objects in an embedded system

**DOI:** 10.1038/s41598-022-27189-5

**Published:** 2023-01-05

**Authors:** Mingi Kim, Heegwang Kim, Junghoon Sung, Chanyeong Park, Joonki Paik

**Affiliations:** 1 Department of Artificial Intelligence, Chung-Ang University, 84 Heukseok-ro, Seoul, 06974 Korea; 2 Department of Image, Chung-Ang University, 84 Heukseok-ro, Seoul, 06974 Korea

**Keywords:** Computational science, Computer science

## Abstract

Recent advances in deep learning realized accurate, robust detection of various types of objects including pedestrians on the road, defect regions in the manufacturing process, human organs in medical images, and dangerous materials passing through the airport checkpoint. Specifically, small object detection implemented as an embedded system is gaining increasing attention for autonomous vehicles, drone reconnaissance, and microscopic imagery. In this paper, we present a light-weight small object detection model using two plug-in modules: (1) high-resolution processing module (HRPM ) and (2) sigmoid fusion module (SFM). The HRPM efficiently learns multi-scale features of small objects using a significantly reduced computational cost, and the SFM alleviates mis-classification errors due to spatial noise by adjusting weights on the lost small object information. Combination of HRPM and SFM significantly improved the detection accuracy with a low amount of computation. Compared with the original YOLOX-s model, the proposed model takes a two-times higher-resolution input image for higher mean average precision (mAP) using 57% model parameters and 71% computation in Gflops. The proposed model was tested using real drone reconnaissance images, and provided significant improvement in detecting small vehicles.

## Introduction

Deep learning-based object detection is widely used in intelligent visual surveillance systems. Recently, deep object detection using a low-power embedded system is gaining increasing attention due to widespread smartphones and unmanned aerial vehicles (UAVs). Although state-of-the-art object detection models have recorded a significantly improved accuracy, the detection accuracy of small objects is usually lower than that of large objects. Detecting small objects, such as traffic signs acquired by a vehicle camera and a person acquired by a drone, is an open problem in object detection tasks. Major reasons for the difficulty in detecting small objects using a light-weight model include:

1. The number of pixels representing a small object is so small that there are no sufficient features to be learned.
2. The feature information of a small object may be offset by that of large objects. Since deep learning-based object detection network is learned through convolution layer, semantic information is extracted while overlaying the network, which has a Encoder–Decoder with a U-Net structure to reduce the size of feature maps while increasing the number of channels. Therefore, it is highly probable to lose feature information on a small object.
3. A light-weight model usually takes a low-resolution input image to reduce the computational complexity, which makes small object detection difficult. For example, if a full-HD image of size 1920 $\times$ 1080 has a small object of size 1616, the size of the small object become 55 in the resized image of size 640,640.
4. It is unrealistic to apply various small object detection methods in a single network. In particular, most industrial applications requires a light-weight network in an embedded environment.

In this paper, we propose an efficient method to improve the performance of small objects detection by solving the above-mentioned problems. We first present an efficient method that handles high-resolution images to improve the detection performance of small object, and then present a sigmoid fusion method that overcomes the difficulty in learning small small object with few-pixel information. The proposed method used YOLOX as the baseline network^1^ with an anchor-free detection model.

## Related work

### Small object detection

Deep learning-based small object detection is one of the challenging tasks in the fields of computer vision because recognition of a small object requires a sufficiently high resolution image, which increases the computational load. Various convolutional neural network (CNN)-based object detection methods have recently been proposed in the literature. The well-known networks include a two-stage detector using region proposal called regions with convolutional neural networks (RCNN)^2^ and one-stage anchor-based detectors called YOLO series^3–5^. However, these methods are not suitable for small object detection since their accuracy is guaranteed only when an object is sufficiently large. Variants of object detection methods were proposed to solve the small object detection problem.

Using a convolutional backbone network, we can extract a higher-level feature map containing information of a small object at the cost of losing a lower-level feature map containing spatial information. To compensate for the trade-off, various methodologies have been proposed to combine the shallow feature with the deep feature^6–13^. These approaches can make the deeper layer contain a sufficient amount of spatial information, which is helpful in detecting small objects.

The number of datasets containing small objects is much smaller than that of large objects. Even in the same dataset, the number of small objects in each category is not sufficient for balanced training. To compensate for an insufficient number of small objects, data augmentation techniques, which can artificially increase the number of data, play an important role in training small objects^14,15^.

Finally, only anchors with a high intersection of union (IoU) score are regarded as positive samples, and the rest of them are negative. Small objects are often classified as negative samples because they are not likely to overlap with ground truth due to the small size. Therefore, the data imbalance problem arises because the number of negative samples are much more than that of positive samples. To solve this problem, several methods have been proposed to adjust weights based on the pre-trained machine learning model so that positive and negative samples have similar numbers^6,16–20^. In addition, various types of loss functions have been proposed to reset weights between unbalanced positive and negative samples for each epoch when training the network^21,22^.

### Context information

Both global and local context information are widely used to improve the performance of small object detection. In general, we can extract context information from both surrounding and spatial location information of the object. The context information is especially necessary to detect small and occluded objects with incomplete object shape information.

#### Local context information

To detect a small object, it is necessary to consider the neighbouring area of the object. Local context information plays a role in representing a visual context of the surrounding region of the object to be detected. Zagoruyko et al. proposed a multi-path network (MPNet) that includes a trained classifier, and utilizes four contextual regions consisting of a foveal structure^23^. In addition, Zeng et al. proposed a bi-directional CNN (GBDNet) that extracts features from multi-scale contextualized sub-regions surrounding the object to improve the detection performance^24^. Li et al. proposed a novel attention to context CNN (ACCNN) to improve the object detection performance using both global and local context information^25^. Zhu et al. proposed a fully convolutional network called *CoupleNet* with two branches, one of which captures the information of the local portion of the object, and the other encodes the global context information with ROI pooling^26^. Guan et al. proposed a new semantic context aware network (SCAN) that includes a local fusion module that builds segmented feature maps using top-down flow and lateral connection and context-aware feature maps by applying multiple pooling operation^27^. SCAN combined context and precise location information to improve the object detection performance for occluded or small objects.

#### Global context information

Global context takes the overall structure in the image into account to learn from the scene-level context. Li et al. proposed a new pooling method with either row- or column-wise max pooling by introducing a global context module using a separate convolution kernel^28^. Bell et al. proposed an inside-outside network (ION) that uses both internal and external information of the ROI^29^. The ION captures context information outside the ROI using the spatial current natural network. Chen et al. proposed WeaveNet which extracts context information from adjacent scales, and repeatedly weaves for more sophisticated context reasoning on multiple scales^30^. DeepDNet proposed by Ouyang et al. used the classification score as a contextual feature for small object detection, and connects it with the object detection score^31^. Li et al. proposed AC-CNN that uses several multi-stacked long short-term memory (LSTM) layers to capture the global context^25^. Zhu et al. proposed SegDeepM that utilizes both existing segmentation and global context to improve the performance of small object detection^32^.

## Proposed method

We present a light-weight deep learning model for small object detection to obtain conflicting objectives, *lighter, faster*, and *better*, at the same time. Our prior study presented most affecting factors on the performance of small object detection, which can be supported through various experimental results. We designed the object detection network on the premise that the model should be implemented in a real-world embedded environment, which has limited computational capabilities. In that context, any sophisticated methods with large computational costs due to a large number of kernels and global average pooling cannot be implemented in an embedded environment, and the corresponding inference time may exponentially increases. To solve that problem, we present two plug-in modules: (1) high-resolution processing module (HRPM) and (2) sigmoid fusion module (SFM), considering the embedded environment. The HRPM efficiently processes high-resolution image to learn feature information of small objects, and the SFM reduces mis-classification errors due to a limited number of pixels of a small object by concentrating weights on small object features. This paper presents an efficient object detection model that processes high resolution image by applying the proposed modules.

### Prior study

Although there are various methods to improve the performance of an object detection model, there are three approaches that can improve the performance without changing the existing model structure:

1. Depth-scaling makes the network learn more powerful semantic information by stacking the layers of the Baseline model deeper,
2. width-scaling increases the number of channels in each layer, called width, to learn more rich feature information, and
3. resolution-scaling increases input resolution, where more pixel information can be used to learn more information about objects.

The method of scaling three factors is called the model-scaling^33,34^. Recent research argued that the method of improving performance by combining three factors results in the highest accuracy. In addition, there is a model scaling method considering the amount of computation^35^. To determine which factor most affects small or tiny object detection, we selected YOLOX^1^ as the baseline with an anchor-free model. We used VisdroneDet-2019 dataset for all performance evaluation, which is widely used for performance evaluation of small object detection^36^.

Table 1 shows the performance of baseline models with a number of parameters that are determined by the depth and width of convolution kernels. It can be seen that the larger the size of the model, the higher the performance is obtained. The parameters and Gflops of YOLOX-m and YOLOX-l differ more than twice, but the performance improvement is insignificant.

**Table 1:** Performance of baseline models according to the number of parameters.

Model	Input_size	Parameters (M)	Gflops	mAP_50	mAP_5095
YOLOX-s	640	8.94	26.65	0.321	0.189
YOLOX-m	640	25.29	73.53	0.342	0.205
YOLOX-l	640	54.15	155.35	0.348	0.216

Table 2 shows the performance of YOLOX-s according to the input resolution when the depth and width of the model is fixed. The mAP significantly increases as the resolution increases.

**Table 2:** Performance of YOLOX-s according to the input resolution.

Model	Input_size	Parameters (M)	Gflops	mAP_50	mAP_5095
YOLOX-s	640	8.94	26.65	0.321	0.189
YOLOX-s	896	8.94	52.24	0.371	0.219
YOLOX-s	1280	8.94	106.62	0.406	0.239
YOLOX-s	1536	8.94	153.53	0.416	0.249
YOLOX-s	1920	8.94	239.89	0.447	0.265

Comparing Tables 1 and 2, YOLOX-s (Input_size 1536) has similar Gflops to YOLOX-l (Input_size 640), but has six times smaller number of parameters with higher accuracy in the sense of mAP_50 and mAP_5095. In addition, compared to YOLOX-l (Input_size 640), YOLOX-s (Input_size 896) has significantly lower Gflops and Parameters, but has higher performance. Based on that observation, it is natural to say that the input resolution is the most important factor to determine the performance of small object detection. Small objects with a small number of pixels are difficult to learn, because they do not have a sufficient amount of learnable feature information. In addition, small object detection requires both spatial edge and robust semantic information. However, if the image is down-sized, a certain amount of pixel information is lost, and learning ability of the object is limited. On the other hand, if the model takes a high-resolution image as input, the learning feature information increases at the cost of increased complexity, computational load, and inference time. We use FLOPs and Parameters to measure the computational load and model complexity, respectively,

1 $$\begin{aligned} \text {Flops} & = k^2 w^2 r^2 d,\\ \text {Parameters}& = k^2 w^2 d , \end{aligned}$$

where *k* represents the number of kernels, *w* the number of channels, *r* the size of resolution, and *d* the depth. Flops, which determines the computational load or inference time, is defined as Parameters multiplied by squared resolution, $r^2$, as in (1). Therefore, increasing the resolution without any processing can lead to a large amount of computation. Based on the prior study, we found that the most efficient way to increase the performance of small objects is to increase the input resolution. In this paper, we propose a module that minimizes computation while learning feature information about small objects as much as possible while taking a high-resolution image as input. In particular, we propose a light-weight module that improves the performance of small object detection and has a small computation volume so that it can be implemented even in an embedded environment. The modules proposed in this paper are as follows.

### High resolution processing module

**Figure 1:**
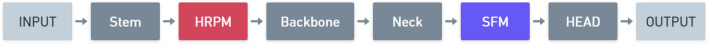
Overall architecture of the proposed model.

Receiving high resolution images as input is followed by a large increase in computation. To learn the information of learnable objects in high resolution images effectively and prevent an significant increase in computation, we propose an efficient module called High Resolution Processing Module (HRPM). In that context, HRPM processes high resolution images in a simple and effective manner. The HRPM is located immediately after the stem module which the input image is through as shown in Fig. 1. Therefore, HRPM is located in the position needed to handle a large amount of Tensor, so it is necessary to design a simple and efficient module. The HRPM is placed in front of the backbone network to learn the edge and color information of the image learned at the shallow level. HRPM amplify spatial information that can be learned at the shallow level quickly and efficiently in high resolution images.

**Figure 2:**
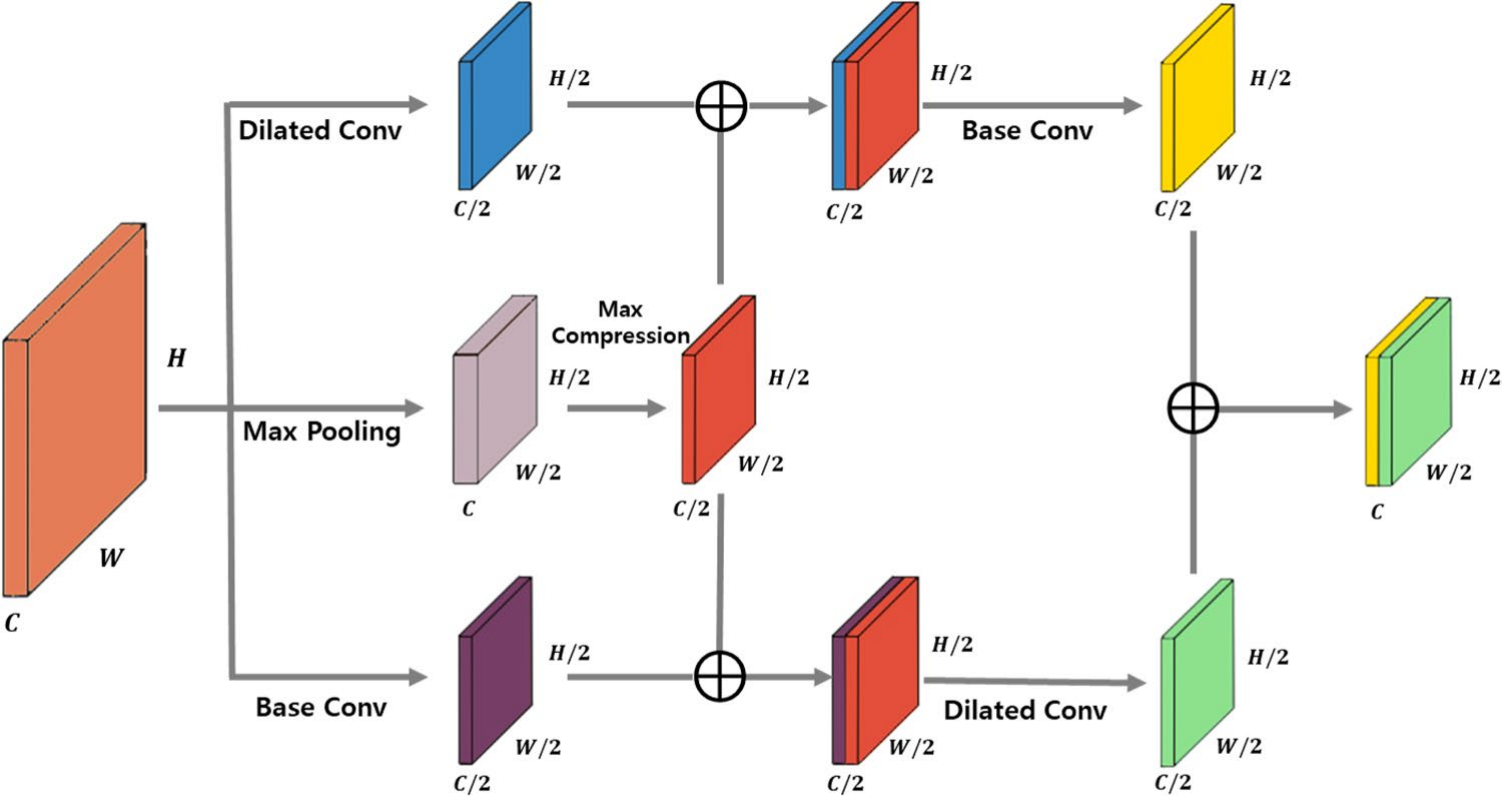
High-resolution processing module (HRPM).

Figure 2 shows the structure of the HRPM. The HRPM is divided into three paths, each learning information that fits its role.

#### Learning Local context information using dilated convolution

Small object detection requires context information due to the lack of information on the object itself. In particular, local context information refers to a context around an object to be detected. This provides effective information for small object detection. On the first path of HRPM, local context information of small objects is learned through dilated convolution^37^. The higher the resolution image, the greater the amount of tensor to be processed. Therefore, local context information is learned while simultaneously halving HWC through dilated convolution.

**Figure 3:**
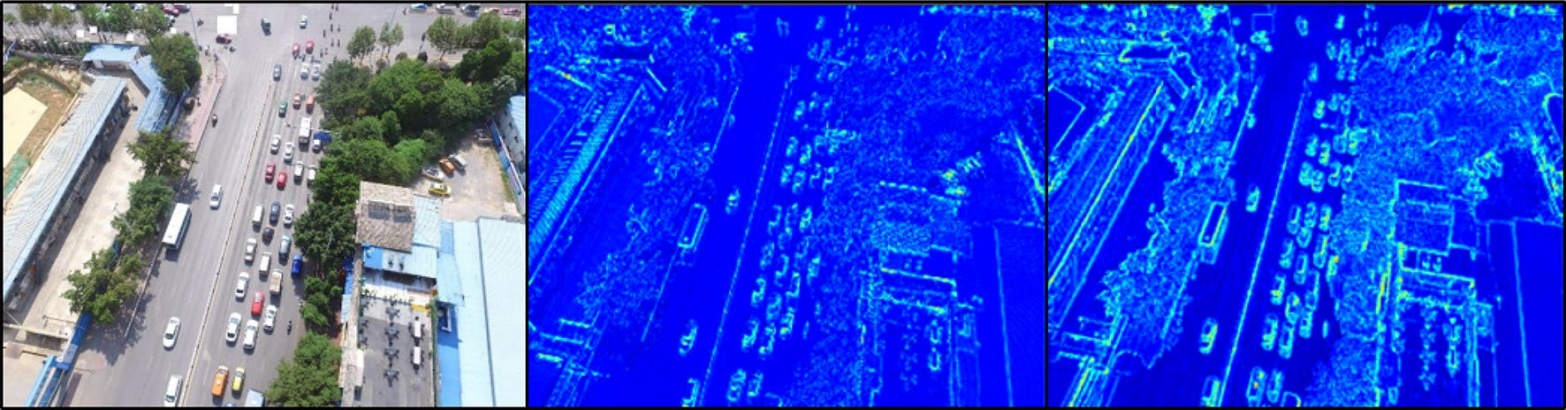
Feature map visualization image comparing base and dilated convolution, left: original input image, middle: base convolution, right: dilated convolution.

Figure 3 visualizes the feature map showing the difference between dilated convolution and base convolution. All visualizations of feature map presented in this paper represent the average value for the entire channel by normalizing the value of the tensor through the Min-Max Scaler. As can be seen from Fig. 3, dilated convolution learns more spatial information by increasing the receptive field. While reducing the amount of computation, it expands the learnable area. As a result, context information around a small object is learned. In addition, since it is possible to take a large amount of receptive field without using pooling operations, the loss of spatial dimension is alleviated and computational efficiency is elevated. In particular, it is possible to learn the spatial information required for small object detection by maintaining spatial features and is effective in processing high resolution feature maps.

#### Max pooling and max compression

On the second path, the powerful edge information is learned by max pooling and max compression. Among various pooling techniques, most commonly used methods are average pooling and global pooling. However, considering the embedded environment, we should not adopt global pooling, which is inefficient in computation. Average pooling is more suitable for small object detection than max pooling, because feature information of small objects is likely to be offset through max pooling. However, the reasons for adopting max pooling rather than average pooling in this paper are as follows. Since HRPM is located in front of the backbone network and learns the edge of the background as well as all objects in high resolution images, it learns together with the edge of the small object. In addition, large size of max pooling is not used because we aim to implement it in embedded environments. Therefore, we model the HRPM to learn edge information efficiently using max pooling of small size of kernel to obtain refined high resolution input image and robust edge information.

**Figure 4:**
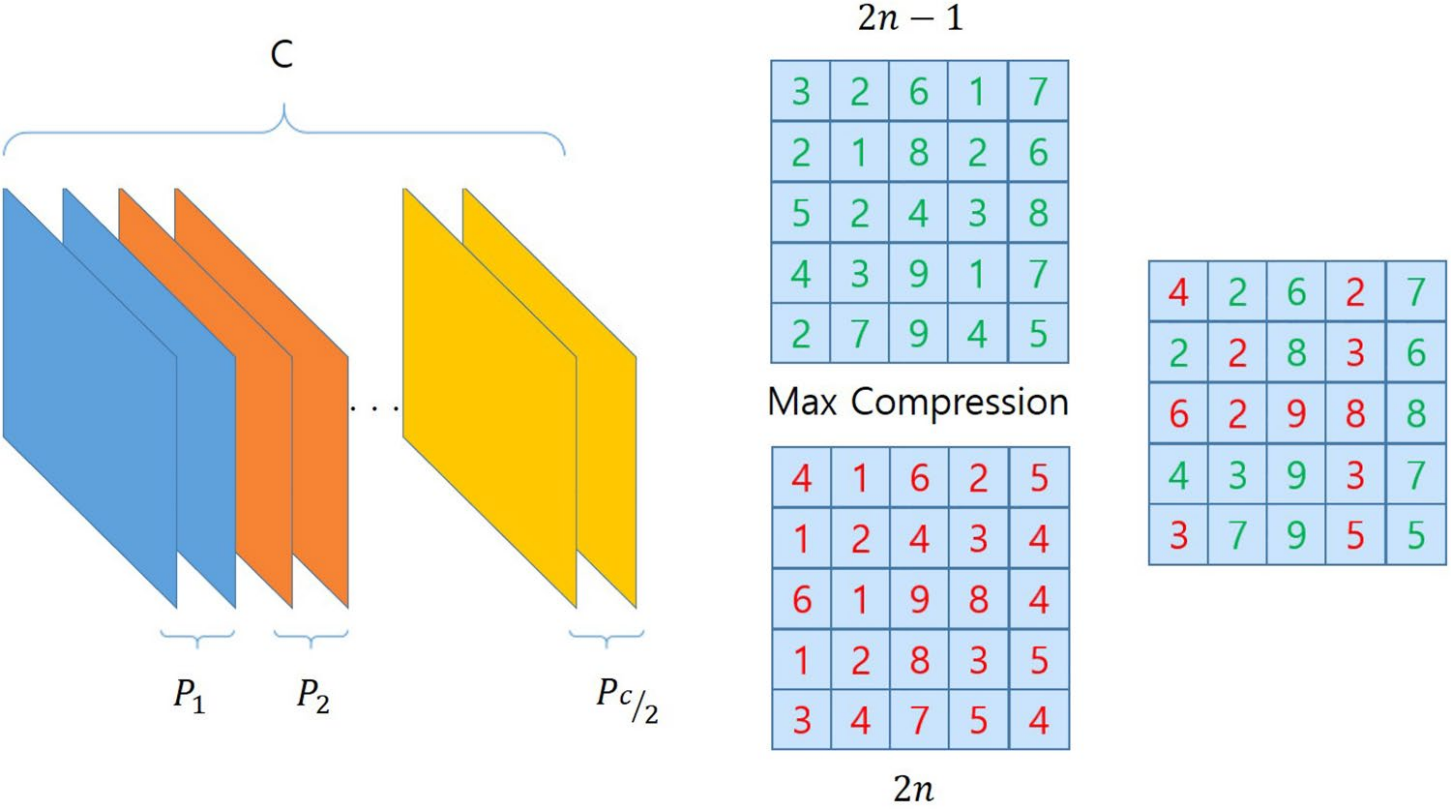
Max compression.

The following max compression pairs the feature maps from max pooling by channel, as shown in Fig. 4. Max compression has two roles. First, only the information of the strong Edge is integrated. Feature maps from max pooling learn various edges for each channel. However, feature information learned at the shallow level is stronger for the spatial edge information than Semantic information. Therefore, the information uniquely learned in each channel is also edge information. HRPM can learn more efficient edge information by integrating strong edge information through max compression.

**Figure 5:**
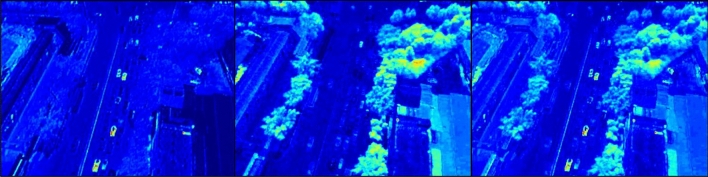
Visualization of max compression features.

As shown in Fig. 5, it can be seen that the feature map is created by max compression of two channels into one channel. Second, HRPM which processes the large amount of tensor makes the entire network be light-weight.

At this time, channels containing necessary information through max compression are selected and the remaining channels are removed to derive computational efficiency and speed. In particular, faster computational speed can be derived by reducing the exponentially increased output tensor volume due to using HRPM.

#### Multi-scale integration and light-weight module

As shown in Fig. 2, dilated convolution learns local context information, max pooling and max compression learn powerful edge information, and base convolution learns feature information of objects. Feature information through the max compression is concatenated into the local context information and feature information. Subsequently, base convolution for learning feature information of an object is performed in the feature map containing local context information, and dilated convolution is performed in the feature map containing feature information, so local context information and feature information are learned on the multiple scale. The change in INPUT and OUTPUT tensor size through HRPM is the width and height of the feature map, and the number of channels is the same. In addition, since we model HRPM as a plug-in, it is designed to have a structure that does not affect the internal structure of the model. When a high resolution image is received as an input, the information taken from the high resolution through HRPM is learned quickly and effectively by amplifying the spatial information of the object. In addition, for implementation in embedded environments, HRPM is designed with simple and small parameters, and a model with low computational capacity despite receiving high resolution images as input.

### Sigmoid fusion module

In small object detection, there is a problem that pixel information is insufficient and background noise is easily confused with object information. Especially in a light-weight model with fewer parameters, there is a difficulty due to the lack of powerful semantic and spatial information of small objects. Therefore, small objects which are difficult to detect have a low confidence score and a high possibility of mis-classification. In this paper, we propose a module that alleviates the mis-classification of objects by focusing the spatial information of the object through the Sigmoid Fusion Module (SFM). The SFM is located between the PAFPN^11^ and HEAD as shown in Fig. 1. In addition, there is no change in the feature map size of INPUT and OUTPUT, and we model SFM in the form of plug-in. Each of the three OUTPUTs passing through PAFPN has a different feature map size, which is utilized to detect small, middle, and large objects.

**Figure 6:**
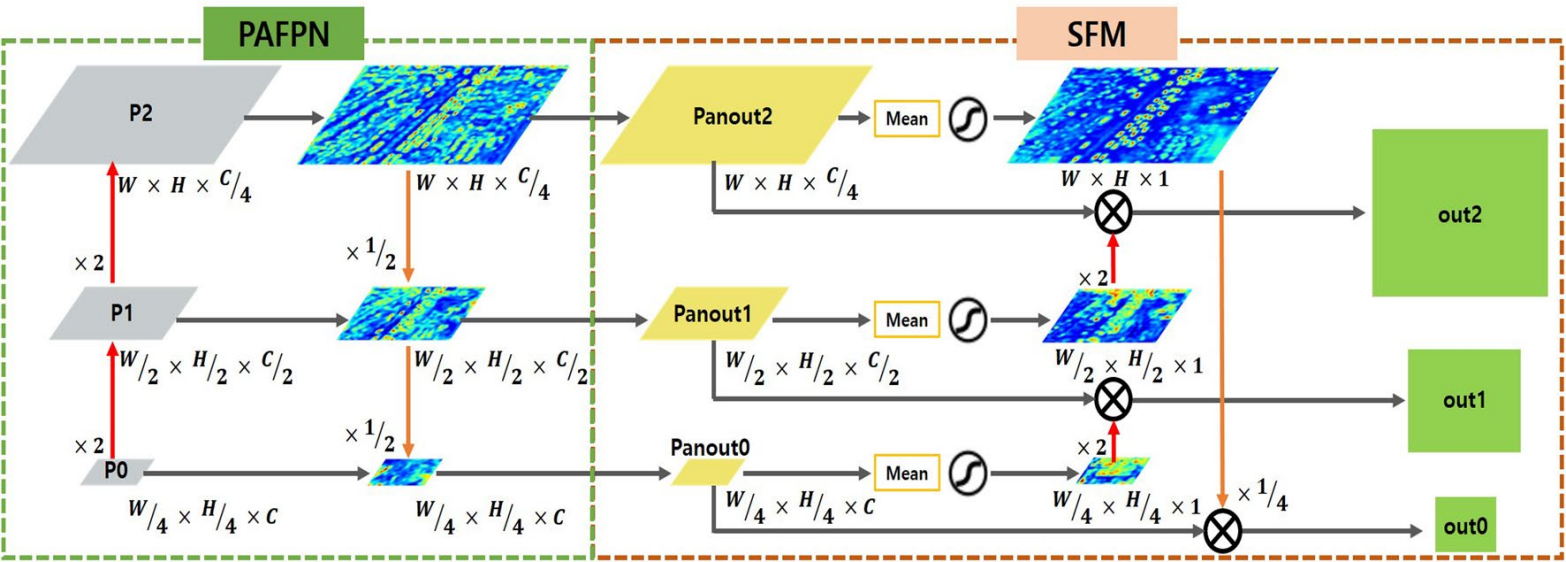
Sigmoid fusion module (SFM).

Figure 6 shows the structure of the SFM, which does not have a layer to learn, and is designed to be performed only by simple computation. The SFM is as follows. PAFPN has robust results on the various scale by fusing the spatial feature information with the shallow level and semantic information with the high level. Conversely, SFM fuses only the spatial information of the high level and the shallow level, compensates lost spatial features for small objects and penalizes mis-learned spatial features for background.

Once the feature map passes through mean and sigmoid operations, attention map consists of only 1 channel is out as a result, which is multiplied by the input feature map to amplify the spatial information in all channels. The input feature map learns different feature information from each channel. Each learned information remains intactly, and makes a relative difference from noise by adding weight to the information on the object. As a result, it has an effect of being more concentrated on objects. In addition, it can be seen that the object that is mis-classified has a low weight by fusing the feature map of different levels. The reason is that the higher the feature level, not only has strong semantic information, but has powerful feature information strong in noise.

**Figure 7:**
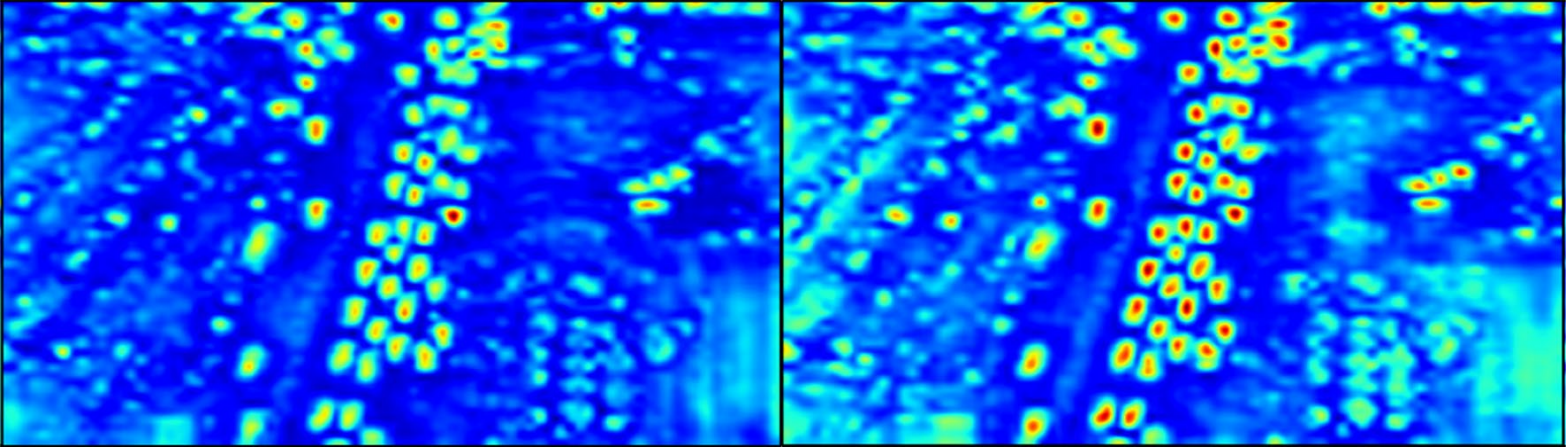
Visualization of SFM features: (left) with SFM and (right) without SFM.

Figure 7 shows the visualization of the feature map of SFM compared to the model with only HRPM applied without SFM applied. It is clear that the addition of SFM is more focused on the object than on the original.

In this paper, we model a light-weight network for small object detection by processing high resolution images over HRPM. We also use SFM to reduce mis-classification of small objects and help the model focus high-weight feature information. The following sections show various comparative experiments between the original YOLOX^1^ and YOLOX applying the proposed method.

## Experimental results

### Experimental setup

The experiments in this paper were implemented in RTX 3090 (24G) environments. Comparative experiments of the YOLOX^1^ applied with the two modules and the original YOLOX were learned and evaluated under the same conditions. Visdrone2019-DET^36^ was used as the dataset. Since this paper aims to implement in an embedded environment, it has been learned and evaluated with a light-weight models with a small computation amount and parameters. Table 3 shows the experimental detail parameters.

**Table 3:** Experiment details.

Training hyper-parameter	Argumentation parameter
Warm up_epochs = 5	Mosaic_prob = 1.0
Max_epochs = 100	Mixup_prob = 1.0
Min_lr_ratio = 0.05	hsv_prob = 1.0
EMA = true	Flip_prob = 1.0
Batch_size = 8	Translate = 0.1
Weight_decay = 0.9	Shear = 2.0

### Comparison with baseline model

Table 4 compares our model, which applies HRPM and SFM to YOLOX-tiny with the original YOLOX-s model. As shown in Table 4, our network (Input_size 1280) has twice larger input than the YOLOX-s (Input_size 640). However, because of HRPM and SFM, it performs better despite smaller number of parameters and Gflops. Through HRPM, our model learns the feature information of small objects that appears at high resolution, and avoid exponentially increasing computations. In addition, the above results were shown by focusing the weights on the objects again through SFM. As the computation amount can be efficiently reduced by applying it to a model through the module presented in this paper, it can be sufficiently used even in a low power embedded environment with limited computation amount. Table 5 shows the difference between applying SFM and not doing it. It can be seen that the detection accuracy is improved by reducing the mis-classification of small objects through SFM.

**Table 4:** Comparison of our network and YOLOX-s.

Model	Input_size	Parameters (M)	Gflops	Precision	Recall	mAP_50	mAP_5095
Our network (YOLOX-tiny + HRPM + SFM)	1280	5.05	18.92	0.228	0.450	0.332	0.192
Original Network (YOLOX-s)	640	8.94	26.65	0.219	0.418	0.321	0.189

**Table 5:** Ablation study on our network with Input_size 1280.

Model	Precision	Recall	mAP_50	mAP_5095
Our network (HRPM with SFM)	0.228	0.450	0.332	0.192
Our network (HRPM without SFM)	0.195 (− 0.033)	0.445 (− 0.005)	0.312 (− 0.2)	0.179 (− 0.13)

**Figure 8:**
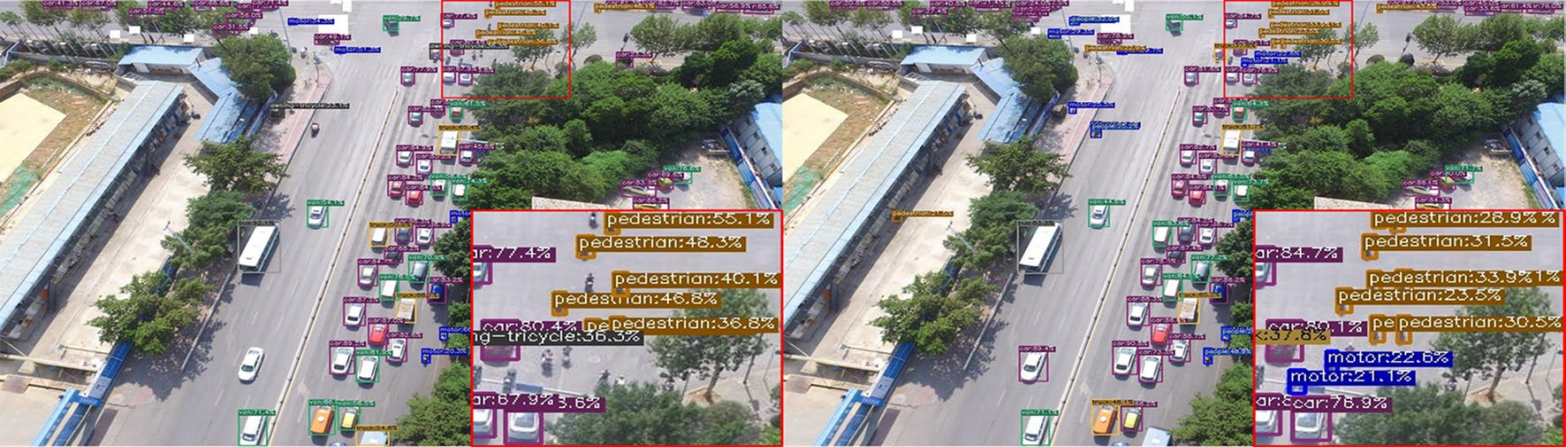
Subjective comparison of small object detection performance: (left) original YOLOX-s model with Input_size 640, and (right) our network with Input_size 1280.

Figure 8 shows the comparative detection result of Our Network (Input_size 1280) and YOLOX-s (Input_size 640) under the same conditions. As the results show, Our Network (Input_size 1280) has more robust results in undetected and mis-classified objects than YOLOX-s (Input_size 640). In particular, tiny size objects such as Pedestrian are confused with noise in the background and are difficult to detect. However, the Our Network (Input_size 1280) to which the presented modules are applied has an accurate result.

**Table 6:** Experimental results of UAVDT.

Model	Test_size	mAP_50	mAP_5095	Parameter	Gflops
Original model	640	0.2580	0.1544	8.94M	26.65
Our network	1280	0.2815	0.1618	5.05M	18.92

To validate the model, Table 6 shows the experimental results using the UAVDT^38^ data set. As shown in the following table, the proposed method achieved higher performance with fewer parameters and Gflops. The result of mAP_5095 confirms that there is a 3% performance improvement.

### Comparison with various object detection model

Table 7 compares the performance of various object detection models. Our model has the largest input and the smallest FLOPs. This means that our network (Input_size 1280) provides similar performance, but has significantly less FLOPs.

**Table 7:** Comparison of performance by model with Visdrone2019-DET Test-dev.

Model	BA	Test_size	FLOPs (G)	Parameters (m)	Recall	mAP_5095	mAP_50
Faster-RCNN^2^	R-50	640 $\times$ 1024	121.1	41.7	21.24	13.19	23.54
YOLOv3-SPP^39^	R-53	640 $\times$ 640	168.7	62.6	20.71	19.25	34.84
RetinaNet^40^	R-50	640 $\times$ 1024	117.4	36.5	16.09	15.81	29.06
FCOS^41^	R-50	640 $\times$ 1024	103.3	10.4	12.96	20.55	38.35
FCOS^41^	R-18	800 $\times$ 1333	140.4	4.5	13.81	22.36	41.14
**Our network**	**CSP-D53**	1280 $\times$ 1280	18.92	5.05	44.99	19.17	33.19

Table 8 is a comparison by one-stage light-weight model with real time that can be implemented in an embedded environment according to the purpose of this paper. Our model has similar FLOPs and Parameters, but has outstanding performance results.

**Table 8:** Comparison of performance by one-stage light-weight detection models with Visdrone2019-DET Val.

Model	BA	Test_size	FLOPs (G)	Parameters (M)	Precision	Recall	mAP_5095	mAP_50
YOLOv5-n^42^	CSP-D53	640 $\times$ 640	4.2	1.77	36.9	28.2	13.1	26.2
YOLOv5-s^42^	CSP-D53	640 $\times$ 640	15.9	7.04	45.2	32.1	17.1	32.1
YOLOv4-s^43^	CSP-D53	640 $\times$ 640	20.5	8.08	29.9	38.2	18.1	33.0
YOLOv4-tiny^43^	CSP-D53	640 $\times$ 640	16.18	5.89	26.8	23.6	11.2	21.9
**Our network**	**CSP-D53**	1280 $\times$ 1280	18.92	5.05	30.2	46.9	22.3	38.2

**Table 9:** Experimental results for 10 classes of Visdrone2019-DET.

Class	Pedestrian	People	Bicycle	Car	Van	Truck	Tricycle	Awning-tricycle	Bus	Motor
Original network	0.2550	0.2078	0.1434	0.6913	0.3471	0.3569	0.2137	0.1916	0.4859	0.3162
Our network	0.2703	0.2070	0.1541	0.7054	0.3545	0.3584	0.2352	0.1855	0.5184	0.3307

As shown in the following Table 9, the proposed model recorded higher performance than the original network for eight classes including Vehicles out of 10 classes. Our model also requires fewer parameters and Gflops than the original model.

**Table 10:** Comparison with the latest small object detection algorithms.

Model	Test_size	mAP_50	mAP_5095	Parameter (M)	Gflops
SF-YOLOv5^44^	640	34.3	18.2	2.24	13.8
Our network	1024	32.4	17.6	5.05	12.11
Our network	1280	38.2	22.4	5.05	18.92

This paper proposes a model specialized for small object detection. Table 10 shows the results of comparison with the latest small object detection algorithms. For a fair comparison, similar Gflops were matched by changing the Test_Size. As a result, the proposed with input size 1024 required a smaller amount of computation in Gflops than SF-YOLOv5^44^ with input size 640, while the performance degradation was negligible. For a better performance, the proposed model with input size 1280 significantly outperforms SF-YOLOv5 with a slightly increased Gflops.

### Experimental results in an embedded system

**Table 11:** Experimental results using Jetson Xavier with Visdrone2019-DET Test-dev.

Model	Test_size	mAP_50	mAP_5095	Parameter (M)	Gflops	Inference time (ms)
Original model	640	0.3163	0.1989	8.94	26.65	91.57
Our network	1280	0.3301	0.2043	5.05	18.92	106.10

Table 11 shows the experimental results in Jetson Xavier. Experimental results record high performance with fewer parameters and Gflops. Although the inference time was slightly increased, the difference is trivial considering that the input size increases by four times. This result proves that the performance of the proposed method operating on an embedded device is the same as that of a general GPU. Finally, since the inference time is approximately 0.1 s, the proposed method can process 10 frames per seconds, which is close to the real-time processing speed.

**Figure 9:**
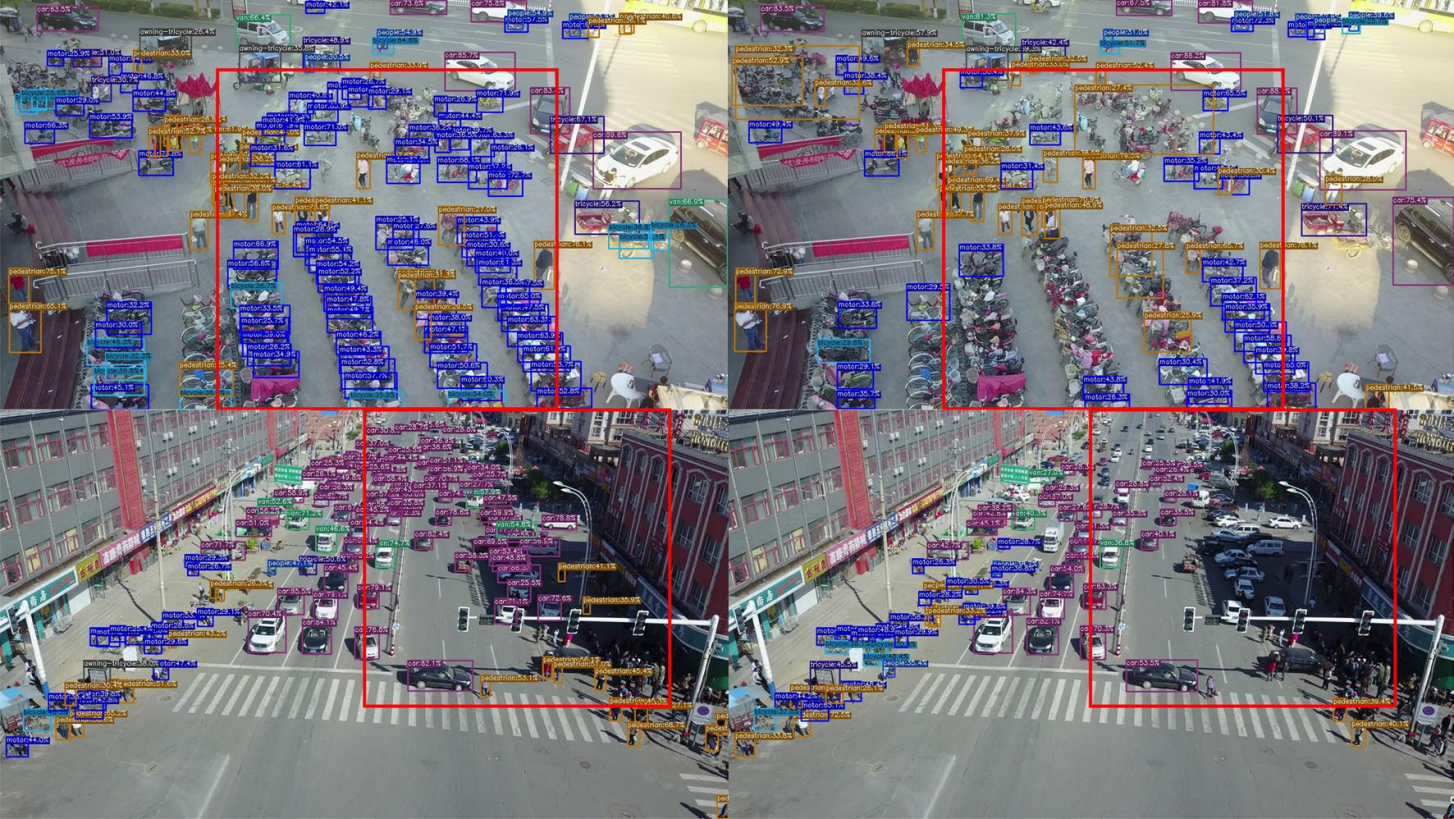
Subjective comparison of small object detection performance: (left) our network with Input_size 1280, and (right) YOLOX-s with Input_size 640.

**Figure 10:**
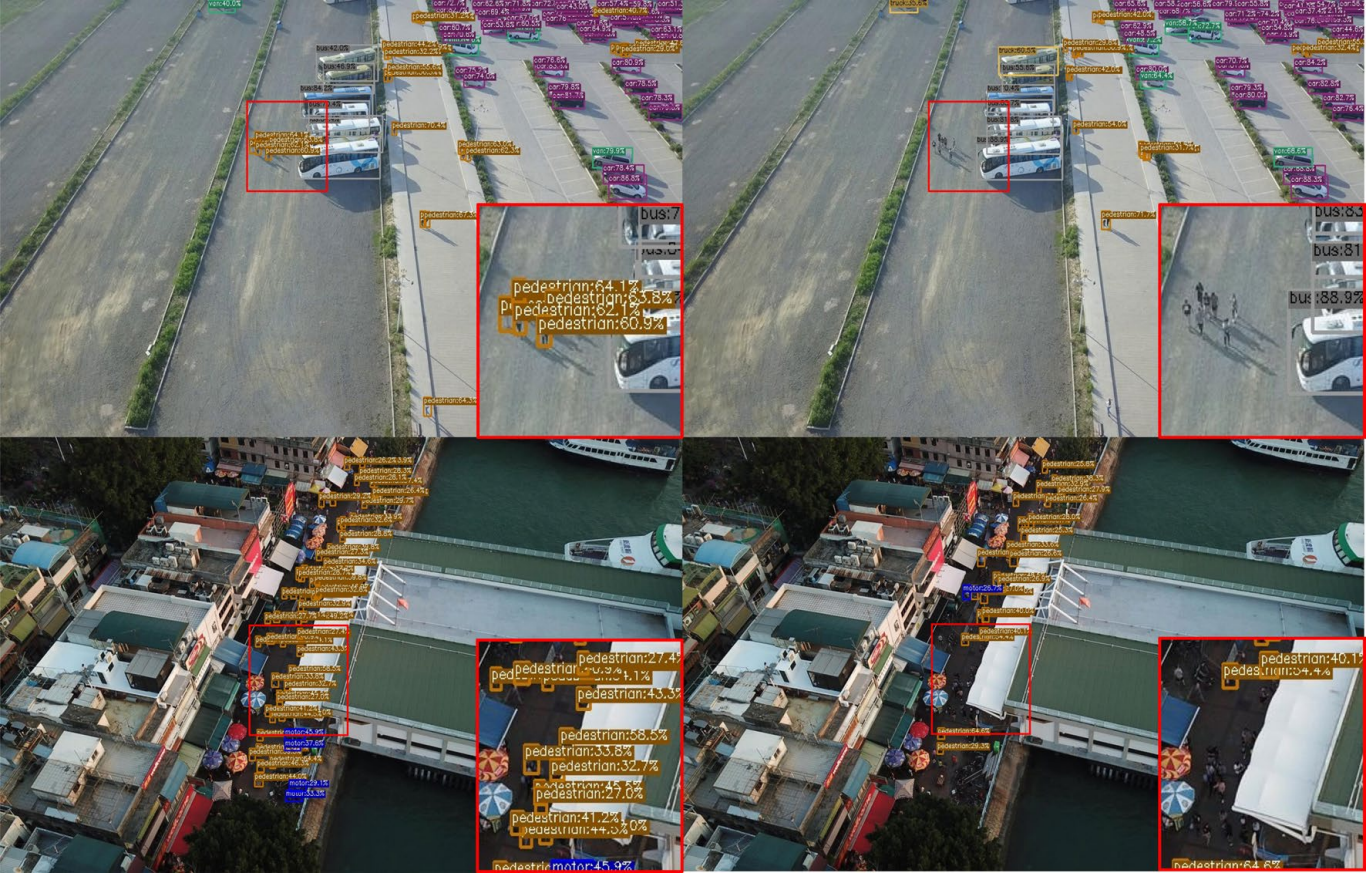
Subjective comparison of small object detection performance: (left) our network with Input_size 1280, and (right) YOLOX-s with Input_size 640.

Additional experimental results are shown in Figs. 9 and 10.

## Conclusion and further work

We proposed high-resolution processing module (HRPM) that efficiently processes high-resolution images to improve the performance of small objects detection, and also proposed sigmoid fusion module (SFM) that alleviates mis-classification errors caused by insufficient pixel information when small objects are learned. The HRPM learns local context information of small objects that can be extracted at high-resolution, and compresses increased channels. The SFM fuses multiple features with weights using the sigmoid function. The proposed model combining the HRPM and SFM realizes a light-weight, accurate detection of small objects. The proposed method recorded a significantly improved performance than existing light-weight model such as YOLOX with fewer parameters and Gflops. Specifically, the proposed model produces higher Recall, which is a measure the performance of small object detection. The proposed method can be applied in a low-power embedded system in various industrial environments. In the future, we will provided plug-in functions of both HRPM and SFM for easy deployment in a real embedded system.

## Data Availability

All data generated or analysed during this study are included in this published article.
